# Unveiling the Hidden Challenges: Non-Motor Disorders in Parkinson’s Disease

**DOI:** 10.3390/brainsci13121710

**Published:** 2023-12-12

**Authors:** Francisco Nieto-Escamez, Esteban Obrero-Gaitán, Héctor García-López, Irene Cortés-Pérez

**Affiliations:** 1 Department of Psychology, University of Almeria, 04120 Almeria, Spain; 2 CIBIS Research Center (Centro de Investigación para el Bienestar y la Inclusión Social), University of Almeria, 04120 Almeria, Spain; 3 Department of Health Sciences, University of Jaen, Paraje Las Lagunillas s/n, 23071 Jaen, Spain; icortes@ujaen.es; 4 Department of Nursing, Physical Therapy and Medicine, University of Almeria, Road Sacramento s/n, 04120 Almeria, Spain; hector.garcia@ual.es

Parkinson’s disease (PD) is not just a motor disorder, it is a complex condition that affects every aspect of a patient’s life, from cognitive impairment and psychiatric disturbances to autonomic dysfunction and sleep disturbances [1]. Recognizing and addressing the non-motor aspects of PD is crucial for improving the overall well-being and quality of life for those living with the disease. Recent advances in our understanding of these non-motor disorders, and the development of innovative treatment approaches, offer hope for a better future for PD patients [2].

While the exact cause of Parkinson’s disease remains the subject of extensive research, emerging evidence suggests a significant role of neurotoxic compounds in its pathogenesis [3]. Understanding the intricate relationships between neurotoxic compounds and PD is crucial to unraveling its origins and developing effective strategies for its prevention and treatment. Thus, in one of articles included in this Special Issue, Ilie et al. [4] observed that rotenone, a dopaminergic antagonistic that crosses the blood–brain barrier (BBB) and directly enters the central nervous system, and also other agents like valproic acid, levodopa/carbidopa, a probiotic supplement of lactobacillus, or a mixture of these compounds, impact zebrafish behavior, particularly in terms of sociability and aggression. However, probiotics had a positive impact, reducing the aggressive behaviors. Even the control group showed some behavioral impairments, although they were less pronounced than in the experimental groups. This research, focusing on animal behavior, marks the initial stage of a potentially promising research direction, particularly regarding the understanding of aggression and social dysfunction, which could have implications for PD patients. The study’s approach, involving immunohistochemistry and assessing neuroinflammation and antioxidant enzyme impairment, holds substantial potential for further insights into this area.

Microglial cells are implicated in the progressive loss of dopaminergic neurons in PD through the release of potentially harmful substances. Excessive microglial phagocytosis of dopaminergic neurons in the substantia nigra is a key pathological event in PD [5]. In their article, Tada et al. [6] studied the impact of zonisamide (ZNS) on mitochondrial reactive oxygen species in microglial cells in a mouse PD model induced by two neurotoxins, MPTP and LPS. ZNS was found to inhibit the phagocytic activity and mitochondrial reactive oxygen species generation in LPS-treated microglial cells, potentially making it an effective antiparkinsonian drug that protects neurons in inflamed PD brains. These findings suggest that ZNS may modify the risk of rapid PD progression by influencing mitochondrial effects on microglial dysfunction.

Among the challenges in managing PD is addressing the fluctuations in the effectiveness of levodopa, a key medication used to alleviate motor symptoms. To tackle this issue, Opicapone, a third-generation catechol-O-methyltransferase (COMT) inhibitor, prolongs the half-life of levodopa and maintains stable plasma levels of the drug, emerging as a promising therapeutic option [7]. In a groundbreaking multicenter, prospective study, Santos García et al. [8] evaluated the impact of Opicapone on non-motor symptoms (NMS) in PD patients. This research, conducted in Spain, focused on the global NMS burden in PD patients treated with Opicapone. Opicapone demonstrated remarkable efficacy in reducing the NMS total score, particularly in domains related to sleep, fatigue, mood, gastrointestinal symptoms, and pain. Notably, Opicapone was well-tolerated and safe, with a drug maintenance rate exceeding 90% after six months of treatment, even though dyskinesia was the most common adverse event reported. This study sheds light on Opicapone’s potential as a therapeutic option for managing both motor and non-motor symptoms in PD, offering hope for improved patient outcomes and a better quality of life.

Psychiatric disturbances, such as depression, anxiety, and psychosis, are common in PD. These NMS not only contribute to the overall burden of the disease, but can also exacerbate motor symptoms [9]. Depression, for example, can lead to increased physical disability and decreased response to dopaminergic therapy [10]. Therefore, identifying and addressing these psychiatric symptoms is essential for improving patients’ well-being and overall outcomes. In the study by Janssen-Aguilar et al. [11], the authors aimed to investigate the clinical and sociodemographic factors associated with major depressive disorder (MDD) in PD patients in a neurological referral center in Mexico. While the severity of MDD was higher in females than in males, there were no statistically significant differences between them. Marital status and educational level did not show a significant association with MDD severity. The study also explored the prevalence of arterial hypertension as a potential risk factor, and examined the relationship between rigidity, gait disturbances, and MDD. Patients with moderate to severe MDD exhibited more rigidity at the onset of PD, fewer gait alterations, and a higher prevalence of left-side disease onset. Additionally, cognitive assessments revealed mild cognitive impairment, with females scoring lower on the MMSE. These findings shed light on the complex factors contributing to MDD in PD patients and underscore the importance of considering these variables in clinical practice and research.

In recent years, transcranial sonography (TCS) has seen increased use as a supplementary tool for diagnosing PD by examining brainstem and subcortical structures [12]. Del Toro-Perez et al.’s [13] systematic review delves into ultrasound findings related to NMS in PD patients. The authors emphasize a link between brainstem raphe (BR) hypoechogenicity and depressive states in PD, potentially indicating structural BR disruption. They also note that substantia nigra (SN) hyperechogenicity is associated with a higher risk of depression, and may serve as a marker for PD development. The co-occurrence of SN hyperechogenicity and BR hypoechogenicity is connected to a history of depression preceding PD. Furthermore, the study explores the role of the vagus nerve in PD progression, with varying results in its relation to NMS, while also acknowledging the limitations of ultrasound evaluation protocols and the examination process.

Sleep disturbances in PD encompass a wide range of complexities, posing challenges in the development of clear treatment recommendations [14]. With the exception of rapid eye movement sleep behavior disorder (RBD), which can manifest as an early symptom, many sleep disorders tend to emerge in the later phases of the disease, frequently evading detection by affected individuals. This underscores the difficulty in effectively addressing these conditions within the framework of PD management. In Lauretani et al.’s [15] article, the authors detail sleep-related issues linked to PD, available medications, and the current status of treatment. The authors emphasize the need to consider sleep disturbances in PD patients from a multifaceted standpoint. It is crucial to recommend therapeutic interventions that align with the disease’s stage, especially when dealing with elderly patients. These recommendations should be carefully weighed alongside other medications and the patient’s existing health conditions to prevent potential risks like sedation and other detrimental side effects.

The term vascular parkinsonism (VP) is one of the most controversial in neurology, given the heterogeneity of the clinical picture that defines it. VP has also been termed “lower body parkinsonism”, because it can manifest as predominant parkinsonism of the lower extremities, with difficulty walking, the absence of tremors, and minimal or no response to levodopa treatment. The studies reviewed by Del Toro-Pérez et al. [16] suggest that VP is a heterogeneous entity that should be properly subclassified to identify those patients with a response to levodopa. On the other hand, new therapies such as vitamin D, repetitive transcranial magnetic stimulation (rTMS), and intracerebral transcatheter laser photobiomodulation therapy (PBMT) warrant further studies to demonstrate their efficacy.

Virtual Reality has been utilized alongside medication in Parkinson’s Disease treatment, offering the flexibility to tailor intervention plans by adjusting the content, duration, intensity, and feedback as required [17]. García-López et al. [18] discuss the use of non-immersive virtual reality (NIVR) in the rehabilitation of patients with PD to improve balance and reduce the risk of falls. These authors have conducted a systematic review that includes ten studies, highlighting the positive impact of NIVR on static and dynamic balance, along with a decrease in fall rates. This work also mentions that NIVR appears to be more effective than conventional physical therapy, and can be combined with other therapeutic methods to enhance results. However, the studies exhibit certain limitations, such as sample size variations and a lack of blinding, and therefore more research is needed in this area. Overall, this article concludes that NIVR has emerged as a promising tool in PD rehabilitation, offering potential benefits for balance and fall prevention.

Gastrointestinal (GI) issues can manifest in the initial stages of PD and, in certain instances, may appear years before motor symptoms [19], significantly impacting patients’ quality of life [20]. Specifically, gastroparesis plays a role in the malnutrition and weight loss frequently observed in PD patients. In their study, Soliman et al. [21] explore the underlying physiological changes contributing to gastrointestinal dysfunction in PD. They also delve into the clinical symptoms associated with impaired stomach movement, particularly gastroparesis, outlining the diagnostic criteria for this condition and discussing its contemporary management based on recent findings. The authors suggest that correlating histological findings, assessments using novel endoscopic techniques, and treatment outcomes could assist in tailoring personalized treatment approaches.

Subclassifying PD based on NMS offers a promising and more precise approach for individualized treatment. Recent research suggests that apathy may serve as a specific indicator of a non-motor subtype known as the Park Apathy subtype [22]. Apathy manifests itself in all disease stages, and may serve as a prodromal symptom in some [23]. In their article, De Waele et al. [24] highlight the intricate pathophysiology of apathy in PD, indicating that distinct neural networks contribute to separate dimensions of apathy. They suggest that the LARS questionnaire can assess these dimensions, potentially aiding in personalized therapy, since each dimension corresponds to specific neurotransmitter deficiencies. However, further research is required to understand how these apathy dimensions manifest in PD patients, their progression, and their response to treatment.

NMS in PD encompass ocular, visuoperceptive, and visuospatial impairments linked to the neurodegenerative process. These symptoms include a range of visual issues, such as dry eyes, blink rate reduction, abnormal eye movements, contrast sensitivity and acuity problems, visuospatial challenges, attention difficulties, and perceptual disturbances, often leading to visual hallucinations. Nieto-Escámez et al.’s [25] review aims to provide a comprehensive understanding of these visual disruptions, exploring their neuroanatomical, functional, and neurochemical underpinnings, including the structural and functional changes in cortical and subcortical regions and their connections to neuropsychological findings, while also considering the involvement of various neurotransmitter systems like dopamine, acetylcholine, and serotonin.

In summary, addressing NMS in PD is critical for enhancing patient well-being. The studies in this Special Issue present promising avenues for understanding the disease’s origin and developing effective therapies. These include exploring the impact of different compounds on behavior [4], identifying the role of microglial cells in disease progression [6], and assessing the effectiveness of drugs like Opicapone in managing both motor and non-motor symptoms [8]. Clinical and sociodemographic factors related to psychiatric disturbances are discussed [11]. Del Toro-Pérez et al. [13] show how the advancements in diagnostics like transcranial sonography provide valuable insights, whereas innovative approaches such as virtual reality demonstrate potential benefits [18]. Additionally, the articles by Soliman et al. [21], De Waele et al. [24], and Lauretani et al. [15] describe clinical and neurobiological characteristics of gastrointestinal, emotional, and sleep-related issues, respectively, proposing tailored therapies for each case. Furthermore, Del Toro-Pérez et al. [16] propose a personalized approach by subclassifying VP based on non-motor symptoms. Finally, Nieto-Escamez et al. [25] review ocular and visual impairments, offering a comprehensive understanding of these symptoms and their management. In conclusion, ongoing research efforts are enhancing our understanding of PD’s non-motor aspects, raising hope for improved patient outcomes and a better quality of life.
